# How does dung burial by coprophagous beetles modify the emergence of infectious strongyle nematode larvae? An experimental test across different soil depths

**DOI:** 10.1016/j.ijppaw.2026.101253

**Published:** 2026-06-16

**Authors:** Mecklina Michael Mbundi, J. Grant C. Hopcraft, Gareth P. Hempson, Jason E. Donaldson, Thomas A. Morrison

**Affiliations:** a University of Glasgow, School of Biodiversity, One Health & Veterinary Medicine, UK; b Department of Zoology and Wildlife Conservation, University of Dar es Salaam, P. O. Box 35064, Dar es Salaam, Tanzania; c Nicholas School of the Environment, Duke University, Durham, NC, 27710, USA

**Keywords:** Anthelmintic, Biocontrol, Parasite(s), Larval (L3), Survival, Dung beetle(s), Burial activity, Soil microclimate

## Abstract

During their free-living stage, gastrointestinal nematode (GIN) eggs and larvae may encounter environmental and biomechanical perturbations, such as dung beetle (Coleoptera: Scarabaeidae, including Aphodiinae, Scarabaeinae and Geotrupidae) activity, that could alter their survival. However, dung beetles’ influence in promoting or suppressing nematodes' survival is poorly understood. Past work suggests that the burial of dung underground by dung beetles may either suppress nematode infections, limiting larval emergence above ground, or enhance nematode infections by creating favourable microclimates for eggs and larval development. We examined the effect of simulated burial of wildebeest dung at four soil depths on the density of infectious third-stage larvae (L3) on herbage over a 42-day window in the Serengeti ecosystem. GIN larval density was highest from dung buried at 5 and 10 cm depths below the soil surface, and lowest at 0 cm (on the surface) and 15 cm depths. This suggests that dung beetles may facilitate L3 survival and emergence by creating favourable conditions at shallow depths (5-10 cm), protecting them from disturbances (e.g., desiccation) on the surface. Deeper burial depth (15 cm) by dung beetles, however, appears to suppress the emergence of L3 by creating a physical barrier to upward migration. Across burial depths, L3 density peaked on Day 14 and declined on Days 28 and 42, with no evidence that deeper burial led to a prolonged emergence period. These results reveal dung beetles' complex role in parasite ecology, not merely as suppressors of these free-living stages but also as facilitators of their survival through burial activities that vary with depth. Incorporating this knowledge into pasture and insect management could support the use of dung beetles as potential biological control agents against gastrointestinal nematodes.

## Highlights

• Dung burial depth influences the emergence of infective strongyle larvae.
• Dung beetles' burial activity could either facilitate or suppress parasite emergence.
• Larval emergence onto pastures is greatest at intermediate burial depths (5-10 cm).
• Dung left on the surface (0 cm) and deep burial (15 cm) reduce larval emergence.
• Larval emergence follows a unimodal temporal pattern, peaking at Day 14.

## Graphical abstract

**Figure:**
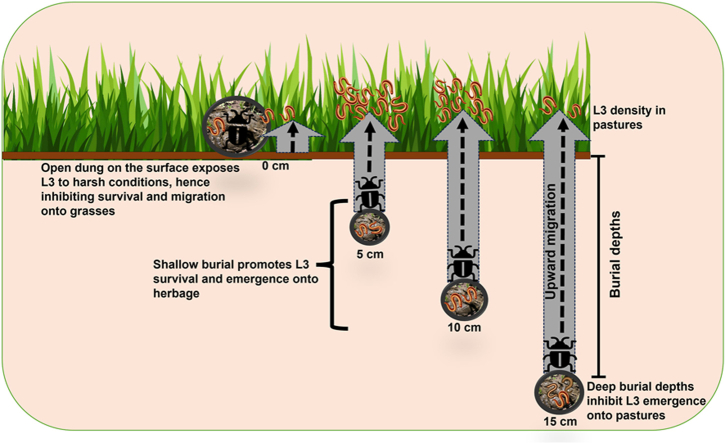

## 1 Introduction

Control of gastrointestinal nematode (GIN) infecting livestock and wildlife has historically relied heavily on anthelmintic treatments targeting the host, due to their affordability and effectiveness (Barone et al., 2020; Roeber et al., 2013; Waghorn et al., 2002). However, the widespread and repeated use of these drugs has contributed to the increase in anthelmintic resistance in parasite populations, despite their initial high efficacy (Beynon et al., 2012; Kaplan and Vidyashankar, 2012; Villar and Schaeffer, 2022). Anthelmintic resistance raises concerns not only for livestock productivity but also for broader ecosystem and conservation outcomes, particularly in systems where wildlife, livestock, and human health interact under a One Health framework (Salvarani et al., 2025). As a result, there has been growing interest in alternative and complementary control strategies that target the free-living stages of helminths in the environment (Chirico et al., 2003; Waller and Faedo, 1996), including biological control agents such as nematophagous fungi, soil earthworms, and dung beetles (Boughton et al., 2024; Sander and Neumann, 2025; Szewc et al., 2021; Waghorn et al., 2002). However, the experimental evidence supporting dung beetles' role as biological agents in regulating GIN in domestic and wild herbivores remains equivocal, and their contribution may be overstated in assessments of ecosystem services (Forbes and Scholtz, 2024).

Diverse beetle assemblages (Coleoptera: Scarabaeidae, including Aphodiinae, Scarabaeinae and Geotrupidae) play key functional roles in pasture ecosystems, contributing to a wide range of ecological processes such as dung decomposition, nutrient cycling, parasite regulation, seed dispersal, forest regeneration and reduction of carbon emissions (Amraoui et al., 2026). These beetles are broadly grouped into three functional categories: endocoprids (dwellers), which inhabit and feed within dung pats; paracoprids (tunnellers), which excavate soil directly beneath or adjacent to the dung and construct underground brood chambers where dung is transported; and telecoprids (rollers), which form dung into balls and roll them away from the pat before burying them at a separate location for brood or food provisioning (Heinrich and Bartholomew, 1979; Holter and Scholtz, 2007). Among these, tunnellers are particularly important for soil redistribution, as they construct vertical or oblique burrows beneath dung pats, and relocate dung mass into brood chambers typically 20-30 cm below the soil surface (McConnell et al., 2026), although burrow depth can extend much further depending on the species, body size, and soil aeration (Gregory et al., 2015). By contrast, rollers remove portions of dung from the pat, form them into balls and transport them laterally before burial in individually excavated chambers (McConnell et al., 2026). Burial depth in roller species is variable, generally ranging from a few centimetres to around 10-20 cm below soil surface, though some larger species may bury brood balls even deeper in loose soils (Gregory et al., 2015). Through these contrasting behaviours, tunnellers tend to concentrate dung inputs vertically beneath pats, while rollers redistribute dung horizontally and vertically, together shaping patterns of nutrient cycling, parasite suppression, and soil structure modification across systems (Holter and Scholtz, 2007; Stanbrook and King, 2022).

Through dung burial and removal activities, dung beetles have considerable potential as natural biological control agents of both insect pests and GIN in livestock systems (Beynon et al., 2015; Forbes and Scholtz, 2024; Heinrich and Bartholomew, 1979). A range of mechanisms have been hypothesised to control the effect of dung beetles on GIN, although empirical support of these mechanisms is variable and dependent on environmental context. Firstly, by rapidly removing and burying dung, dung beetles alter the spatial distribution of parasite developmental stages. Burial can prevent larvae from successfully returning to the soil surface and surrounding vegetation under conditions where vertical migration is constrained, such as burial depth, low soil moisture, high soil compaction and reduced pore connectivity (Fowler, 2013; Nichols and Gómez, 2014; Sands and Wall, 2017; Schon et al., 2025). Under these conditions, particularly when tunnelling species bury dung at depths of approximately 15 cm or more, infective L3 are likely to experience restricted movement through the soil matrix and reduced ability to migrate back to the surface (Forbes and Scholtz, 2024). This limitation decreases their availability on herbage and the likelihood of host ingestion (Doube, 2018; Forbes and Scholtz, 2024). Secondly, dung beetle activity, particularly by dwellers species, can directly damage parasite eggs and larvae, for example, through incidental ingestion or mechanical disruption during feeding and nesting (Byk and Piętka, 2018; Nichols et al., 2017). Thirdly, dung beetles' tunnelling and fragmentation activities can substantially modify the microenvironment of dung pats by enhancing aeration and acceleration of desiccation, creating unfavourable conditions for the survival and development of GIN larvae (Forbes and Scholtz, 2024). This can limit larval development, reduce larvae's ability to disperse from the dung pat, and ultimately decrease transmission potential to grazing hosts (Nichols and Gómez, 2014). Additionally, dung beetles may indirectly contribute to parasite suppression by facilitating the dispersal of predatory mites and other organisms that feed on nematodes (Edwards and Aschenborn, 1987; Forbes and Scholtz, 2024). Despite these proposed pathways, it remains unclear how dung beetle activity translates into changes in larval availability on herbage, which ultimately determines infection risk (Forbes and Scholtz, 2024). In particular, the role of dung burial depth in mediating these effects has received limited experimental attention under realistic field conditions.

Despite the growing interest in dung beetles as potential biological control agents of GIN, most evidence comes from agricultural systems or controlled experimental conditions, and relatively little is known about how these processes operate in natural ecosystems characterised by highly diverse dung beetle communities and large herbivore assemblages. Understanding these interactions is particularly important in African savannas, where wildlife and livestock frequently share grazing areas and where dung beetles play a major role in ecosystem functioning (Foster, 1993). Dung beetles are highly dependent on dung, and their abundance and activity are closely linked to dung availability (Ahungu et al., 2025; Tonelli et al., 2018). They are also capable of rapidly locating and exploiting ephemeral dung resources through chemical cues (Simmons and Ridsdill-Smith, 2011). In African savanna systems, dung beetle community structure and activity have been shown to closely track large herbivore presence and dung availability, with rapid increases in abundance following wildebeest arrival and declines as herds move on (Foster, 1993; McNaughton, 1985). Although direct evidence of long-distance tracking of migratory herds by individual dung beetle species remains limited, field observations and community-level patterns indicate strong, fine-scale coupling between dung beetle activity and the spatio-temporal movement of migratory herbivores. This suggests that dung beetle assemblages respond dynamically to migration-driven pulses of dung deposition, even if their movement is locally opportunistic rather than coordinated over large spatial scales (Foster, 1993). The dung beetle community in the Serengeti is highly diverse, comprising a full complement of functional groups (rollers, tunnellers, and dwellers), with high species richness typical of intact savanna ecosystems (Ahungu et al., 2025; Foster, 1993). Therefore, this coupling between herbivore movement, dung deposition and dung beetle presence and activity makes the Serengeti an ideal ecosystem for investigating interactions between dung beetle activity and GIN larval survival. However, despite the ecological importance and the strong theoretical basis for their role in parasite suppression, there is limited experimental evidence on how dung burial depth affects the emergence of infective GIN larvae under natural savanna conditions.

Here, we aim to quantify the impact of dung burial by coprophagous beetles on the availability of GIN infective larvae on pastures in a natural savanna ecosystem. Specifically, we examined how burial depth, duration of exposure, and the ambient temperature influence the density of L3 that emerge from dung and migrate onto herbage, where they can be ingested by grazing livestock and wildlife. This migration of infective larvae onto vegetation represents a critical transmission pathway and underpins the quantification of L3 abundance from herbage samples in this study. Based on existing evidence that dung beetles can reduce parasite transmission through dung removal and burial, we hypothesised that burial would suppress L3 availability on pasture by limiting larval migration to the soil surface and exposing larvae to unfavourable conditions. We therefore predicted a negative relationship between burial depth and L3 density, with the lowest larval availability at greater burial depths and the highest at the soil surface, particularly where dung is protected from beetle activity. However, because dung burial may also buffer larvae from surface disturbances that can cause desiccation, it may create more stable microclimatic conditions. Therefore, an alternative outcome is that shallow burial could facilitate larval survival relative to surface dung. This raises the possibility that burial effects are not strictly linear but instead vary with depth depending on the balance between protection from environmental stress and constraints on larval movement.

## 2 Materials and methods

### 2.1 Study area

We conducted this study at the Serengeti Wildlife Research Centre based in Seronera, Serengeti National Park, Tanzania, located at latitude S2°29′ 2.04’’, longitude E34°50′ 39.84’’. The Serengeti is a semi-arid savanna ecosystem, with mean annual rainfall varying between 400 and 1100 mm (Norton-Griffiths et al., 1975; Pennycuick and Norton-Griffiths, 1976). Soils in the Seronera area are predominantly sandy to sandy clay loam in texture (Pallangyo and Emmanuel Mbije, 2021). Approximately 1.36 million wildebeest (*Connochaetes taurinus*), along with 250,000 zebra (*Equus burchelli*) and 100,000 Thomson's gazelle (*Gazella thomsoni*), track rainfall along a seasonal migratory route through the Serengeti ecosystem (Anderson et al., 2024; Kija et al., 2023). Based on field-derived estimates for large grazing ungulates in the study system, wildebeest produce approximately 10-12 dung pats per individual per day, with variation due to diet, season and activity (Hempson G., personal communication, 2024). Using an average dung mass of ∼300 g per pat (Kimaro et al., 2025), this translates to an estimated system-wide input of approximately 4000–4900 tonnes of dung per day. Dung deposition could be a major source of environmental contamination of GIN eggs, potentially altering the risk of GIN infection for migratory and non-migratory resident hosts (Donaldson et al., 2024; Kimaro et al., 2025). Therefore, the annual migration of herbivores, particularly wildebeest, generates extreme spatial and temporal heterogeneity in dung deposition, creating pulsed resource availability across the Serengeti Ecosystem (de Visser et al., 2015).

### 2.2 Experimental design

**Fig. 1:**
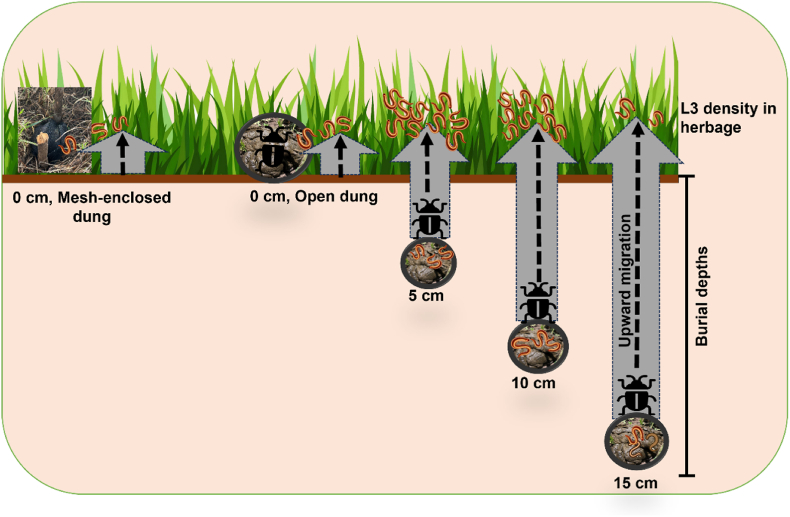
Conceptual framework and experimental design illustrating how dung burial depth influences the availability of infective gastrointestinal nematode larvae (L3) on pasture. Dung containing a standardised number of GIN eggs/larvae was placed either on the soil surface (0 cm) or buried at increasing depths (5, 10, and 15 cm), simulating dung relocation by different dung beetle functional groups. The figure outlines two alternative mechanisms: (i) burial may suppress L3 availability by physically restricting larval movement to the pastures, and (ii) burial may enhance L3 survival by buffering larvae from desiccation and creating a more stable microclimate. These contrasting mechanisms predict different relationships between burial depth and L3 density on herbage.

To evaluate the effect of dung burial by coprophagous beetles on the density of GIN larvae onto grass, we set up controlled experimental cages of 3 × 3 m. Dung burial treatments and L3 pasture sampling were conducted within the central 2 × 2 m area inside the cage to reduce edge effects (Kimaro et al., 2025). The study was replicated across five blocks, each initially containing four caged exclosures (Appendix Figure S2), which were tested in two experiments (January-February 2024 and November-December 2024). For the second experiment, a fifth cage was added to each block (Appendix Figure S3) as a control, in which dung placed on the soil surface was fully covered by mesh, preventing dung beetle activity (Appendix Figure S4), and bringing the total to 25 cages. Within each block, cages were spaced at least 10 m apart. Experimental cages were established and enclosed with chicken-wire fencing approximately 2-4 weeks before dung application to exclude grazing and prevent any new dung deposition during the pre-experimental period. Before the start of the experiment, all cages were thoroughly cleared of existing dung and surface organic debris to minimise background contamination. This preconditioning ensured that any emerging L3 larvae originated from the experimentally applied dung rather than pre-existing field inputs. Each cage was assigned a specific dung burial depth treatment. Dung containing a standardised number of GIN eggs was either placed on the surface (0 cm) or buried at 5, 10 or 15 cm (Fig. 1). These depths were selected to reflect the range of dung relocation behaviours exhibited by different functional groups of dung beetles. Surface dung (0 cm) represented conditions in the absence of burial, and surface dung was accessible to all functional groups, but especially dwellers (Endocoprids). Shallow burial (5 cm) reflected the burial activity of smaller tunnelling species (Paracoprids) and small rollers (Telecoprids) that relocate dung just below the soil surface, whereas intermediate depths (10 cm) reflected typical burial depths of medium-sized tunnellers and large rollers. Deep burial (15 cm) represented the burial activity of larger tunnelling species capable of relocating dung to greater soil depths (Edwards and Aschenborn, 1987; Foster, 1993; Ryan et al., 2011).

### 2.3 Preparation of dung for the burial experiment

To standardise the number of GIN eggs for the dung burial experiment, at least 15 fresh wildebeest faecal piles (∼300 g) were collected daily for five consecutive days. On the day of collection (Day 1), faecal egg counts (FEC) were conducted on each dung pile using a modified McMaster method to confirm infection (Hansen and Perry, 1994). Dung piles with high faecal egg counts (>150 eggs per gram of faeces or “epg”) were combined and thoroughly homogenised by hand for each sampling day, then cultured for five days in a dark room under ambient temperature and watered on day two to avoid desiccation of eggs and larvae (Donaldson et al., 2023). After the five-day culture period, dung from each batch was deployed in the field in the evening to establish one experimental block. This process was repeated daily, such that Block 1 was deployed on Day 5, followed by Blocks 2 to 5 on each successive day thereafter. Each block corresponded to dung collected on consecutive days, therefore maintaining a five-day standard culture period across all blocks.

### 2.4 The burial experiment and enumerating the density of GIN parasites

To establish baseline GIN larval pasture counts before the dung burial experiment in the first experiment, all grass within each burial treatment cage was harvested on the morning of Day 5 by clipping grass to a height of 10-15 cm, and samples were placed in labelled plastic bags (Appendix Table S1). In the second experiment, no pasture sampling was conducted on Day 5 because previous work in the system (Kimaro et al., 2025) consistently showed that exclosures remained free of active larvae due to the cleaning protocols used to prevent dung contamination (Appendix Table S2 & Table S3). On the evening of Day 5 for both experiments, hand-rolled dung balls weighing 150 g were created from the homogenised dung samples. Dung balls were randomly assigned to cages and buried at their respective burial treatment depths, with dung in the 0 cm treatment placed on the soil surface. Burial was done manually using a hand trowel, with disturbance restricted to the excavation of discrete holes corresponding to the assigned treatment depths. Following placement of dung balls, excavated soil was carefully backfilled to restore the original soil profile and minimise the disruption of the surrounding structure.

GIN larval counts were conducted by sampling the standing grass on days 14, 28 and 42 to allow sufficient time for the incubated larvae to migrate through the soil profile and onto the grass layer. Infective L3 were recovered from grass samples using a modified Baermann technique (Hansen and Perry, 1994). Clipped grasses were weighed, enclosed in a gauze and submerged in 10-20 L of water, with 2 ml of non-ionic detergent to facilitate larval detachment (Molento et al., 2016). Samples were soaked for 3 h, agitated periodically and left overnight to allow larvae to migrate into the suspension. The following day, samples were rinsed with 1 L of clean water, and the suspension was allowed to settle. The supernatant was then siphoned, leaving approximately 1.5 L of sediments, which were transferred into Baermann funnels and left to settle for 1 h. The recovered sediment was collected, centrifuged at 1500 rpm for 10 min, and the supernatant was discarded in a small volume of water. Lugol's iodine was added to stain larvae before counting. Gastrointestinal nematode third-stage larvae (L3) were identified under a stereomicroscope using the morphological keys of (Friedhoff, 1978; Van Wyk and Mayhew, 2013). Grass samples were subsequently air-dried for 3-4 weeks, and the dry biomass was recorded to standardise larval counts.

### 2.5 Dung removal experiment on the soil surface

To separate the effects of dung removal by beetles from other sources of dung loss (e.g. desiccation, fly activity, and natural decomposition) that could affect parasite survival at the soil surface, a second experiment included a mesh-enclosed surface treatment (0 cm depth) using 1.6 × 1.62 mm mesh size. This treatment consisted of 15 standardised dung balls (150 g each) placed on the soil surface and enclosed within the fine wire mesh to exclude dung beetles while still allowing smaller invertebrates and exposure to ambient environmental conditions. These mesh-enclosed dung balls were compared with open dung on the surface that remained accessible to dung beetles. Dung mass remaining in both open and mesh-enclosed treatments was assessed after two weeks, a period considered sufficient for peak dung beetle attraction and activity decline, allowing estimation of dung removal attributable to beetle activity versus abiotic and microbial decomposition processes. In addition, visible signs of dung beetle activity, including tunnelling, feeding and dung relocation, were systematically recorded throughout the experiment (Appendix Table S4 & Fig. S4).

### 2.6 Measuring environmental covariates

To evaluate the influence of microclimatic conditions associated with burial treatments, near-surface soil temperature was monitored during both experiments, using capacitance-based sensors (ECH20 VP4 series moisture sensors, Meter Group, Inc., USA). Sensors were installed within each caged exclosure, positioned in a corner, with probes inserted shallowly into the soil to ensure adequate soil contact for measuring conditions at the soil surface horizon. Ground temperature was monitored using a thermocouple wire placed above the ground in each cage. Temperature measurements were intended to characterise the environmental conditions experienced by larvae emerging from dung and migrating onto surrounding herbage. Sensors were connected to a data-logging system and programmed to record measurements every 30 min. Daily mean temperature for each cage was calculated by averaging all readings recorded on each pasture sampling day.

### 2.7 Statistical analysis

Data from both experiments were combined to assess the effect of burial depth on L3 abundance (counts) in herbage. Although the experiments were conducted at different times of the year and differed slightly in design (with the inclusion of a day 42 and an additional mesh-enclosed dung treatment in the second experiment), pooling was undertaken to increase statistical power and improve robustness of inference. The mesh-enclosed dung treatment (0 cm burial depth) was analysed separately to evaluate the effect of dung beetle exclusion (Appendix Table S3). As L3 abundance in this treatment did not differ significantly from that of open-surface dung, it was excluded from the main burial-depth analysis to ensure consistent comparisons across burial treatments. The final dataset, therefore, comprised four burial depth treatments (0, 5, 10, and 15 cm) across both experiments, resulting in a total of 100 observations (Experiment 1: n = 40, Experiment 2: n = 60). Sampling was conducted on Days 14 and 28, in both experiments, with an additional Day 42 included in Experiment 2.

To investigate the effects of dung burial depth and sampling day on the abundance of strongyle L3 larvae (count response variable), a negative binomial generalized linear mixed-effects model was fitted using the “g*lmer.nb*” function in the “*lme4*” package (Bates et al., 2015). Burial depth (0, 5, 10, and 15 cm) was treated as a continuous predictor to test for directional trend in larval abundance with increasing burial depth, while sampling day was included as a categorical predictor to account for temporal variation in larval emergence. Model selection was performed using the likelihood ratio test (LRTs) implemented in the “*lmtest”* package. Three competing models were compared: (i) a full model including interaction terms among burial depth, sampling day, and soil temperature; (ii) a model including only the main effects of burial depth, sampling day, and soil temperature; (iii) a simplified model including only burial depth and day as main effects. Model comparisons based on LRTs were used to identify the best-fitting model that best explained variation in L3 abundance.

All models included burial depth, its quadratic term, and sampling day as fixed effects, while ‘Block’ was included as a random effect. To account for the differences in the amount of grass biomass sampled for the L3 recovery across treatments and blocks, log-transformed dry grass weight was included as an offset term in all models. Continuous predictors (soil temperature and burial depth) were scaled to standardise the data and improve model convergence and interpretability. Model fitting was performed using the *"bobyqa"* optimiser with increased iteration limits to ensure model convergence. Overdispersion was checked using the “*performance*” package, and model fit was further evaluated using a simulated residual diagnostic implemented in the “*DHARMa*” package.

To test the effect of dung beetles on the surface dung removal, the proportion of dung removed was modelled as a function of treatment (open dung and mesh-enclosed dung) as a fixed effect, with block as a random effect. Because the response variable (proportion of dung removed) was bounded between 0 and 1 (inclusive), we fitted a generalized linear mixed model with a Beta error distribution using “*glmmTMB”* (Brooks et al., 2017). To account for the effect of treatments (open dung and mesh-enclosed dung both at 0 cm) and soil temperature on L3 density as a count-dependent variable, a negative binomial linear mixed-effects model was fitted using the “g*lmer.nb”* function in the “*lme4”* package (Bates et al., 2015), with *"bobyqa"* optimiser with increased iteration limits to ensure model convergence. Block was included as a random effect, and an offset term for log-transformed dry grass weight (g) was also included in the model (Kimaro et al., 2025). Model assumptions were evaluated using simulation-based diagnostics implemented in the *DHARMa* package, and overdispersion was assessed using the *performance* package. All statistical analyses and visualisations were conducted in R (version 4.5.0).

## 3 Results

### 3.1 Optimal belowground incubation and timing effect on GIN larvae density

**Fig. 2:**
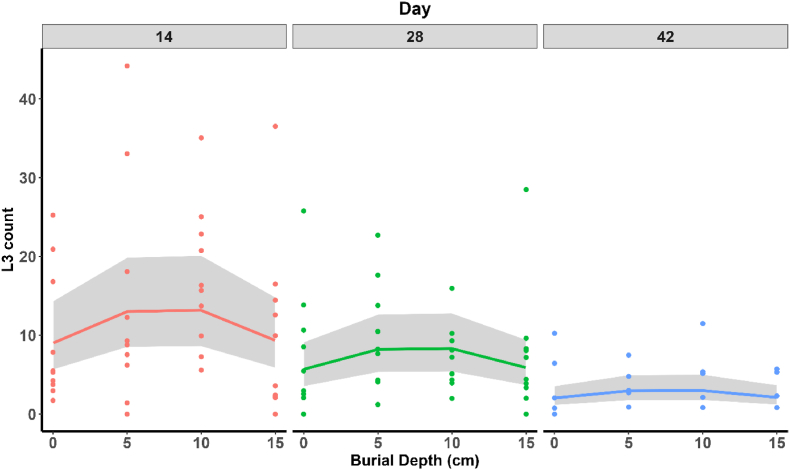
Predicted density of infective stage gastrointestinal larvae (L3) across dung burial depths (0 - 15 cm) and sampling days (14, 28, and 42). Lines represent model predictions derived from the negative binomial generalized linear mixed-effects model, including burial depth (linear and quadratic terms), sampling day, with shaded areas indicating 95% confidence intervals. Points show raw data standardised for sampling effort (L3 count adjusted by mean dry grass weight 69.9 g). Larval counts declined over time and exhibited a nonlinear relationship with burial depth, with the highest larval densities at intermediate depths (5-10 cm) and lower densities at both surface (0 cm) and deeper burial depths (15 cm). Overall larval abundance declined substantially from Day 14 to Day 42.

Dung burial depth had a significant nonlinear effect on L3 abundance. The quadratic term for scaled burial depth was significant (β = −0.225 ± 0.089 SE, *P* = 0.012), whereas the linear term was not (β = 0.014 ± 0.072 SE, *P* = 0.850). This demonstrates a unimodal relationship between burial depth and larval density. Model predictions (Fig. 2) showed that L3 density increased steeply from surface dung (0 cm) to intermediate depths (5-10 cm), where it peaked, and then declined at deeper burial depth (15 cm).

Sampling day had a strong and consistent negative effect on larval density. Relative to day 14, L3 density was significantly lower on Day 28 (β = −0.460 ± 0.157 SE, *P* = 0.003) and further dropped on Day 42 (β = −1.482 ± 0.210 SE, *P* < 0.001: Fig. 2), indicating a pronounced temporal decline in larval availability.

There was no evidence that effect of burial depth varied across sampling days, as the interaction terms were not significant and did not improve model fit (χ^2^ = 0.13, *P* = 0.94; Appendix Table S5a). Soil temperature was also not a significant predictor of L3 density and did not improve model fit (χ^2^ = 0.78, *P* = 0.38; Appendix Table S5b). The final model (Appendix Table S5c), therefore, supports additive effects of burial depth (including a quadratic term) and sampling day as primary drivers of L3 density.

### 3.2 Effects of dung beetle on L3 counts at the soil surface

During the first experiment, approximately 75% of the dung left exposed at 0 cm was removed by dung beetles. In the second experiment, about 85% of the open dung at 0 cm (64 out of 75, based on 15 dung balls per open treatment across 5 blocks) showed tunnelling beneath the dung pats and feeding signs, indicating dung beetle activity (Appendix Figure S4). In contrast, none of the 75 dung balls covered with a mesh-enclosed at 0 cm exhibited any signs of beetle visitation.

**Fig. 3:**
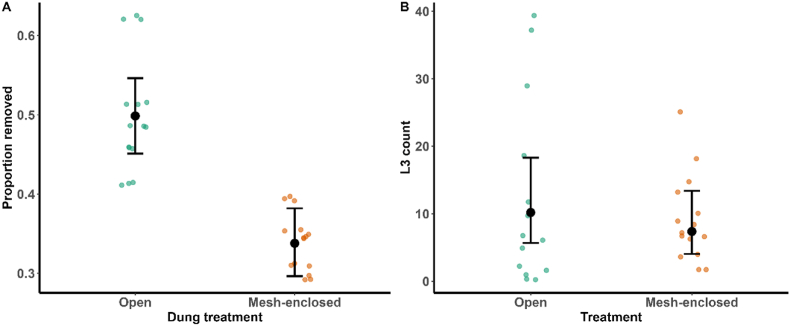
**A)** The proportion of dung removed under open and mesh-enclosed treatments. Points represent raw data (coloured by treatment), while black symbols and error bars indicate model predictions ± 95% confidence intervals from a beta mixed-effects model. **B)** The infective third-stage larvae (L3) density across treatments. Raw data are shown as coloured points, and black symbols represent predicted values ± 95% confidence interval from a negative binomial mixed-effects model, with dry grass weight (DGW) included as an offset and predictions standardised at the mean DGW (82.7 g).

From the second experiment, the proportion of dung removal was significantly higher in the open-surface dung (0 cm), which permitted dung beetle activity, compared to dung enclosed in mesh on the surface, where removal was restricted (β = −0.667 ± 0.035 SE, *P* < 0.001: Fig. 3A). In contrast, there was no statistically significant effect of either treatment (i.e. open dung vs. mesh-enclosed dung), on L3 counts in herbage (β = −0.324 ± 0.266 SE, *P* = 0.224; Fig. 3B). However, the average temperature calculated from temperature recordings taken within each cage, on each pasture sampling day, had a significant negative effect on L3 counts (β = −0.143 ± 0.053 SE, *P* = 0.007: Appendix Figure S1), indicating that L3 recovery from pasture samples declined with increasing temperature.

## 4 Discussion

The most important findings from our study suggest that dung beetles' burial activity promoted L3 survival at shallow burial depths (5-10 cm), which enhanced L3 abundance compared to dung left exposed on the soil surface (0 cm). This pattern suggests that intermediate burial depths provide environmental conditions favourable for GIN larval survival and emergence compared to dung left exposed on the surface. Additionally, L3 emerging from dung that was buried deep (15 cm) was relatively low, suggesting that soil may also pose a barrier for L3 movement at deeper depths. These results indicate that dung burial can either enhance or suppress L3 emergence depending on burial depth. An important implication of our findings, therefore, is that the impact of dung beetles on gastrointestinal parasite infection risk in each location will likely depend on the composition of dung beetle functional groups, particularly the relative dominance of shallow-versus deep-burying species. Ecosystems dominated by shallow-burying dung beetles may therefore experience greater L3 availability on pastures due to the creation of favourable microclimatic conditions at intermediate burial depths, potentially increasing the availability of infective larvae on pastures. In contrast, communities dominated by deep-burying species may contribute to reduced L3 emergence by relocating dung beyond depths from which larvae can effectively migrate back to the soil surface. Consequently, spatial or temporal variation in dung beetle community structure could influence patterns of parasite transmission and grazing in different grazing systems.

### 4.1 Optimal belowground incubation effect on GIN larvae density

The increased density of L3 in this study from dung buried at shallow depth (5-10 cm) suggests that intermediate burial depths create optimal habitat conditions for larval survival and migration (Fowler, 2013). These burial depths likely buffered larvae from environmental extremes while maintaining conditions suitable for larval development and migration to vegetation (Forgie et al., 2018; Waghorn et al., 2002). Our findings align with those of (Waghorn et al., 2002), who reported higher nematode recovery from dung buried at 5 cm compared to surface pats during autumn and spring field trials in New Zealand. Increased recovery of larvae from buried dung, together with their rapid movement from the soil onto herbage, suggests that soil may act as a protective reservoir that supports the survival and movement of infective larvae onto pasture vegetation (Waghorn et al., 2002). Similarly (Fincher and Stewart, 1979), demonstrated enhanced larval migration from dung buried 5-15 cm under high soil-moisture conditions, highlighting the importance of burial-mediated moisture retention. Collectively, these studies support the idea that intermediate burial depths can promote larval survival and migration (Fowler, 2013; Waghorn et al., 2002). However, these benefits appear to decline once burial exceeds the vertical migration capacity of larvae. On the other hand, dung remaining on the surface is more vulnerable to desiccation and physical disturbance, particularly when fragmented by dung beetle activity (Bryan and Kerr, 1989; Chirico et al., 2003; Reinecke, 1960; Sands and Wall, 2017). These conditions can reduce egg hatching success and impair development of free-living larval stages (Forgie et al., 2018; Fowler, 2013; Grønvold et al., 1992). In contrast, dung buried at intermediate depths may provide greater environmental buffering, helping explain the higher abundance observed in our study.

### 4.2 Timing (delay) effect on GIN larvae density

L3 abundance peaked on Day 14, declined by Day 28, and reached its lowest level by Day 42, indicating a strong negative temporal effect on L3 density (Fine et al., 1993). Rather than supporting our hypothesis that deeper burial would delay egg or larval development and migration, thereby prolonging larval availability on pasture, the results suggest that early post-deposition conditions favoured rapid development and migration regardless of burial depth. Similar temporal patterns have been documented elsewhere (Kimaro et al., 2025). reported higher L3 abundance after two weeks compared to later periods (Amaradasa et al., 2010; Fincher, 1973). observed peak larval recovery between weeks two and four following dung deposition, followed by a steady decline. Under wet weather conditions (Sands and Wall, 2017), demonstrated greater L3 migration from faeces colonised by dung beetles during the second week, but reduced by week eight. Together, these findings indicate that larval availability is highest during the first few weeks after dung deposition before progressively declining over time.

### 4.3 Deep burial inhibition effect on GIN larvae density

In contrast to shallow burial depths, deeper burial at 15 cm significantly reduced L3 emergence, providing experimental evidence that greater burial depths can limit larval migration to the soil surface and subsequently reduce pasture contamination under Serengeti conditions. This finding aligns with previous studies demonstrating that burial beyond approximately 15 cm restricts the upward migration of infective trichostrongyle larvae (Gregory et al., 2015; Grønvold et al., 1996; Nichols and Gómez, 2014). Several tunnelling dung beetle species are known to bury dung at or beyond these depths (Cessna, 2017; Forbes and Scholtz, 2024), and reduced L3 recovery has previously been linked to the activity of the deep-tunnelling dung beetles (Forgie et al., 2018). Supporting this mechanism (von Son-de Fernex et al., 2025), using a controlled artificial burial assay, showed that the upward migration capacity of *Cooperia punctata* L3 decreased by 28% with each centimetre increase in burial depth (R^2^ = 0.92). Collectively, these findings indicate that deep-tunnelling dung beetles may play an important role in suppressing gastrointestinal nematode transmission by relocating dung beyond the effective vertical migration range of infective larvae (von Son-de Fernex et al., 2025).

### 4.4 Lack of effects of dung beetle activity on the surface dung on L3 density

At the soil surface, open dung declined in mass more rapidly than mesh-enclosed dung, indicating removal activity by dung beetles (Fig. 3A) (Noriega et al., 2023; Schon et al., 2025; Stanbrook-Buyer et al., 2024). However, despite accelerated dung removal, L3 abundance in herbage did not differ significantly between treatments. One possible explanation is that, in the open dung treatment, dung beetles removed dung and redistributed it into the surrounding soil at unknown and unmeasured depths. Because relocated dung was not tracked, the fate of parasite-containing material and its burial depth could not be determined, representing an important limitation of the study. It is therefore possible that redistributed dung continued to support larval development and subsequent emergence, resulting in similar L3 counts between open and mesh-enclosed dung treatments. Although dung redistribution was not directly quantified in the open treatment, excavation of the buried dung in 5-15 cm treatments showed that dung generally remained undisturbed throughout the experiment, suggesting that redistribution effects were most likely restricted to the open surface treatment. Previous studies have similarly shown that the effects of dung-colonising insects, particularly dung beetles, on L3 are highly context-dependent and strongly influenced by environmental conditions (Sands and Wall, 2017). In our study, temperature had a significant negative effect on L3 counts, indicating that abiotic conditions may have played a greater role in regulating larval survival than dung removal itself. Furthermore, mesh-enclosed dung did not consistently support higher L3 densities despite reduced beetle disturbance. While undisturbed dung can form a crust that limits oxygen diffusion and egg hatching (Chirico et al., 2003; Reinecke, 1960), retained moisture within intact dung pats may also create favourable conditions for larval development under certain conditions. Overall, environmental variability likely moderated treatment effects, contributing to the limited differences in L3 density observed between surface treatments.

## 5 Ecological implications in savanna systems

In savanna ecosystems such as the Serengeti, parasite transmission dynamics are shaped by interactions among diverse herbivore assemblages, species-rich communities of coprophagous dung beetles, shallow heterogeneous soils, and strong climatic and fire variability (Donaldson et al., 2024; Kimaro et al., 2025). Previous studies have documented high dung beetle diversity in the Serengeti Ecosystem, with more than 45 species recorded and a full complement of functional groups present (Ahungu et al., 2025; Foster, 1993). Within this context, dung beetle functional structure is central to helminth dynamics. Historical community data from the Serengeti (Foster, 1993) indicate that dwellers comprised approximately 14-17% of the assemblage, slow and shallow-burying tunnellers 34-58%, fast and deep-burying tunnellers 11-14%, large rollers 11-35%, and small rollers 2-4%. In contrast, recent surveys from the present study system (Mbundi et al., unpublished data) show a community dominated by slow and shallow-burying tunnellers (56.7%), and small rollers (35%), whereas fast and deep-burying tunnellers (5%), dwellers (3.45%), and large rollers (1%). These patterns suggest a substantial shift in functional composition, characterised by an increase in small rollers and a marked decline in dwellers, larger rollers and fast and deep-burying tunnellers.

Functionally, communities dominated by fast and deep-tunnelling species are expected to suppress parasite transmission, whereas slow and shallow-burying assemblages may maintain or enhance larval availability on pastures. Systems dominated by dwellers are likely to be more strongly influenced by environmental drivers than by beetle-mediated effects. Our results show depth-dependent effects of dung burial on helminth dynamics: shallow burial (5-10 cm) enhanced GIN development and migration, while deep burial (≥15 cm) suppressed transmission by restricting larval emergence. However, deep burial is constrained by edaphic conditions. Across much of the Serengeti, particularly short-grass plains used by grazing ruminants during the wet season, soils are shallow and often underlain by hardpan layers near the surface. This likely restricts deep tunnelling and promotes L3 persistence over broad spatial scales. Overall, dung beetle effects on parasite transmission are strongly condition-dependent, emerging from interactions among functional traits, soil structure, climate variability, vegetation, and herbivore movement that together shape infection risk patterns.

## 6 Conclusion and future work

This experimental study shows that dung burial by coprophagous beetles modifies the emergence of infectious strongyle nematode larvae (L3), with effects varying across soil burial depths. Across treatments, L3 emergence was not uniformly suppressed by dung burial activity. Intermediate burial depths (5-10 cm) supported greater L3 emergence than surface dung. In contrast, deeper burial (15 cm) consistently reduced L3 emergence, indicating that relocation of dung beyond the effective vertical migration range of larvae can disrupt transmission pathways. These effects depend not only on burial depth, but also on the functional composition of dung beetle communities. Dung beetles should therefore be considered a functionally diverse group whose effects on parasite transmission vary across ecological contexts. Overall, their role in gastrointestinal nematode management is best considered as part of integrated control strategies rather than as a standalone solution.

Therefore, several extensions for future research should be considered based on this work. Sample sizes in our study were modest, which may have limited our ability to detect certain predicted effects, such as an interaction between burial depth and timing of emergence. It remains unclear whether parasite development time is slowed by burial within dung. Our experiment may not have fully replicated natural dung beetle behaviour in terms of the range of depths and dung ball sizes. For example, some dung beetle species burrow substantially deeper than 15 cm (Nichols and Gómez, 2014) with reported burial depths ranging from 10 to 103 cm (Edwards and Aschenborn, 1987). We therefore recommend that future studies first characterise the composition of natural dung beetle assemblages and then quantify the corresponding distribution of burial depths within those assemblages. While L3 density generally exhibited a peaked relationship with burial depth across sampling days, this pattern was less pronounced at day 42, suggesting potential temporal changes in larval persistence or survival. Although the interaction between day and depth was not significant, there is still a need to consider temporal dynamics when interpreting depth effects. In addition, detailed microclimatic measurements, such as soil moisture and humidity within the grass layer, could provide further insights into how environmental conditions influence the movement and survival of L3 in pasture (Kimaro et al., 2025). Future studies could also incorporate temperature measurements at different dung burial depths to better understand how thermal gradients within the soil profile influence larval development, survival and vertical movement following dung burial. Improved understanding of these processes will enhance predictions of parasite transmission risk under environmental change and inform the use of biological control agents in savanna ecosystems. Furthermore, the effects of burial depth observed in this study may be partly mediated by local soil conditions, including soil texture, pH, microbial activity, and moisture, all of which can influence larval survival and vertical migration through the soil profile. Consequently, the burial thresholds identified in this study may vary among ecosystems and soil types. We therefore recommend that future studies quantify and report detailed soil characteristics to improve comparability across systems and to better understand context-dependent effects of dung burial on parasite survival and emergence.

## CRediT authorship contribution statement

**Mecklina Michael Mbundi:** Conceptualization, Data curation, Formal analysis, Funding acquisition, Investigation, Methodology, Resources, Software, Validation, Visualization, Writing – original draft, Writing – review & editing. **J. Grant C. Hopcraft:** Conceptualization, Formal analysis, Methodology, Resources, Software, Supervision, Validation, Writing – review & editing. **Gareth P. Hempson:** Conceptualization, Formal analysis, Methodology, Resources, Software, Supervision, Writing – review & editing. **Jason E. Donaldson:** Data curation, Methodology, Resources, Writing – review & editing. **Thomas A. Morrison:** Conceptualization, Data curation, Formal analysis, Funding acquisition, Investigation, Methodology, Project administration, Resources, Software, Supervision, Validation, Visualization, Writing – original draft, Writing – review & editing.

## Funding

Mecklina Michael Mbundi acknowledges the United Kingdom Commonwealth Scholarship Commission (CSC) (Sponsor ID: TZCS-2022-539) for funding her PhD studies in Ecology at the University of Glasgow, and the Biotechnology and Biological Sciences Research Council (BBSRC) (Grant Ref: BB/V004484/1) supporting the project “EEID US-UK Collab: Disentangling transport and trophic effects of animal movement on infectious disease&quot;, which made this research possible.

## Conflict of interest

The authors declare that there is no conflict of interest regarding the publication of this paper.
