# Supplementary material for: How does dung burial by coprophagous beetles modify the emergence of infectious strongyle nematode larvae? An experimental test across different soil depths
Source: Int J Parasitol Parasites Wildl. 2026 Jun 16;30:101253. doi: 10.1016/j.ijppaw.2026.101253 (PMC13293723; doi:10.1016/j.ijppaw.2026.101253)
Supplement: Multimedia component 1 [file mmc1.docx]

## **Appendix: Supplementary Materials**


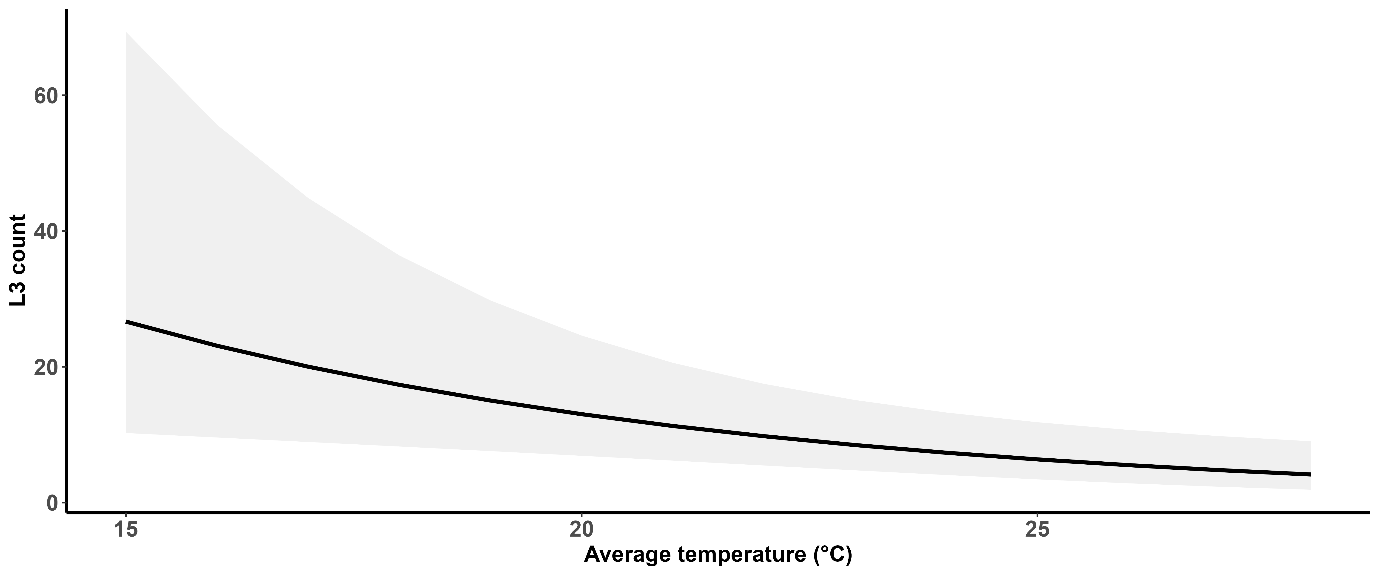


**Figure S1:** Effect of average temperature on L3 count, showing model predictions ± 95% confidence intervals from a negative binomial mixed-effects model, with dry grass weight held constant at its mean value (82.7g).


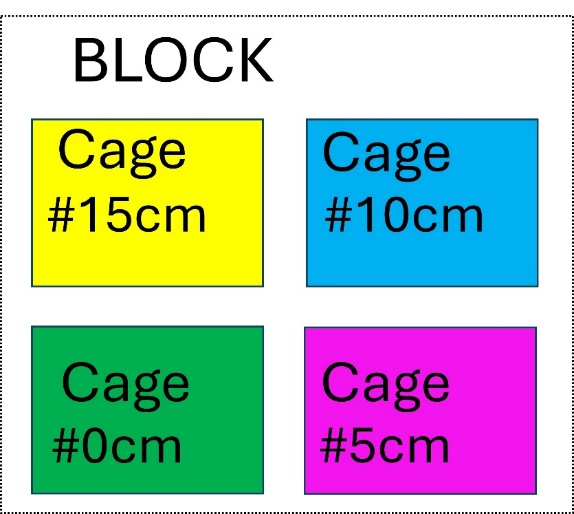

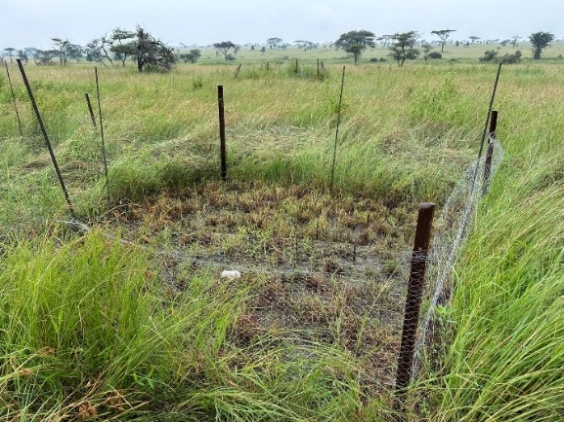


**Figure S2:** Block layout of four caged exclosures (each assigned a distinct burial depth treatment) during experimental period 1, alongside a photograph of a burial treatment cage in Serengeti National Park. Photograph ©Mecklina Michael Mbundi.


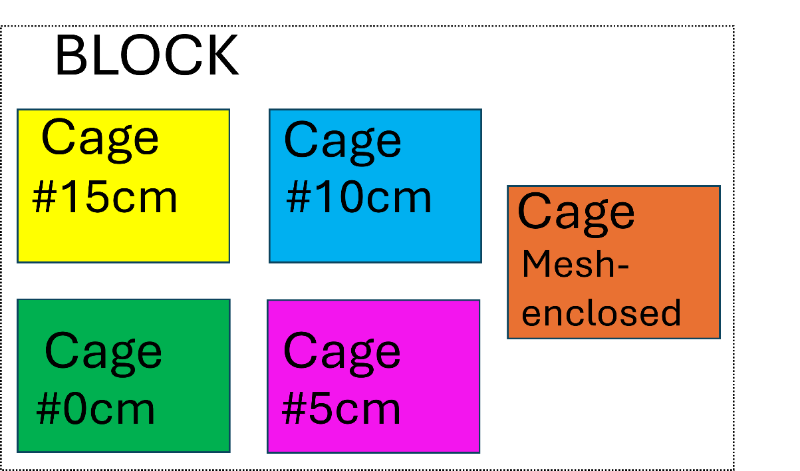

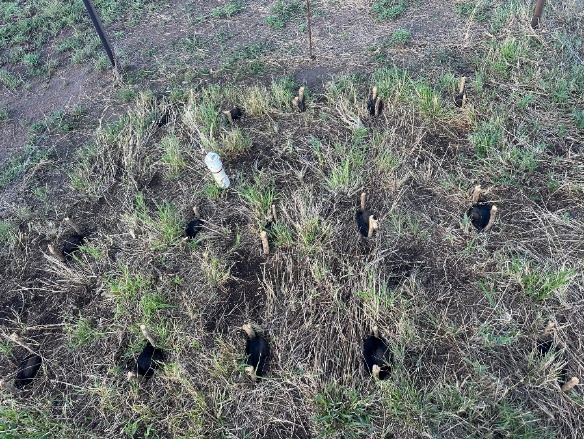


**Figure S3:** Layout of five caged exclosures within a block during Experiment 2, with each cage assigned a specific treatment, alongside a representative photograph of a control exclosure where dung was enclosed with fine amesh netting to restrict dung beetle access. Photograph ©Mecklina Michael Mbundi.


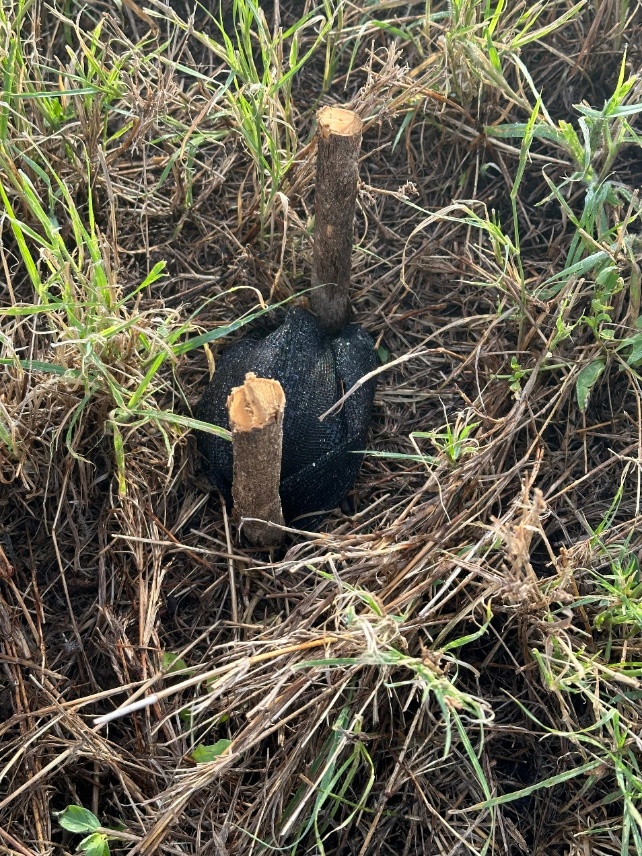

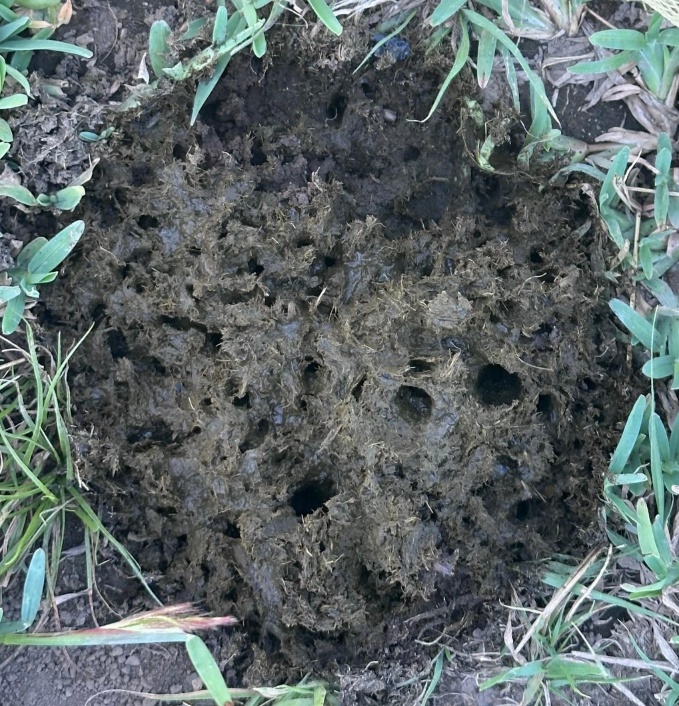


**Figure S4:** Mesh-enclosed and open dung pats showing evidence of dung beetle tunnelling activity. Photograph ©Mecklina Michael Mbundi

**Table S1:** Summary of burial treatments applied in the first experimental period

**
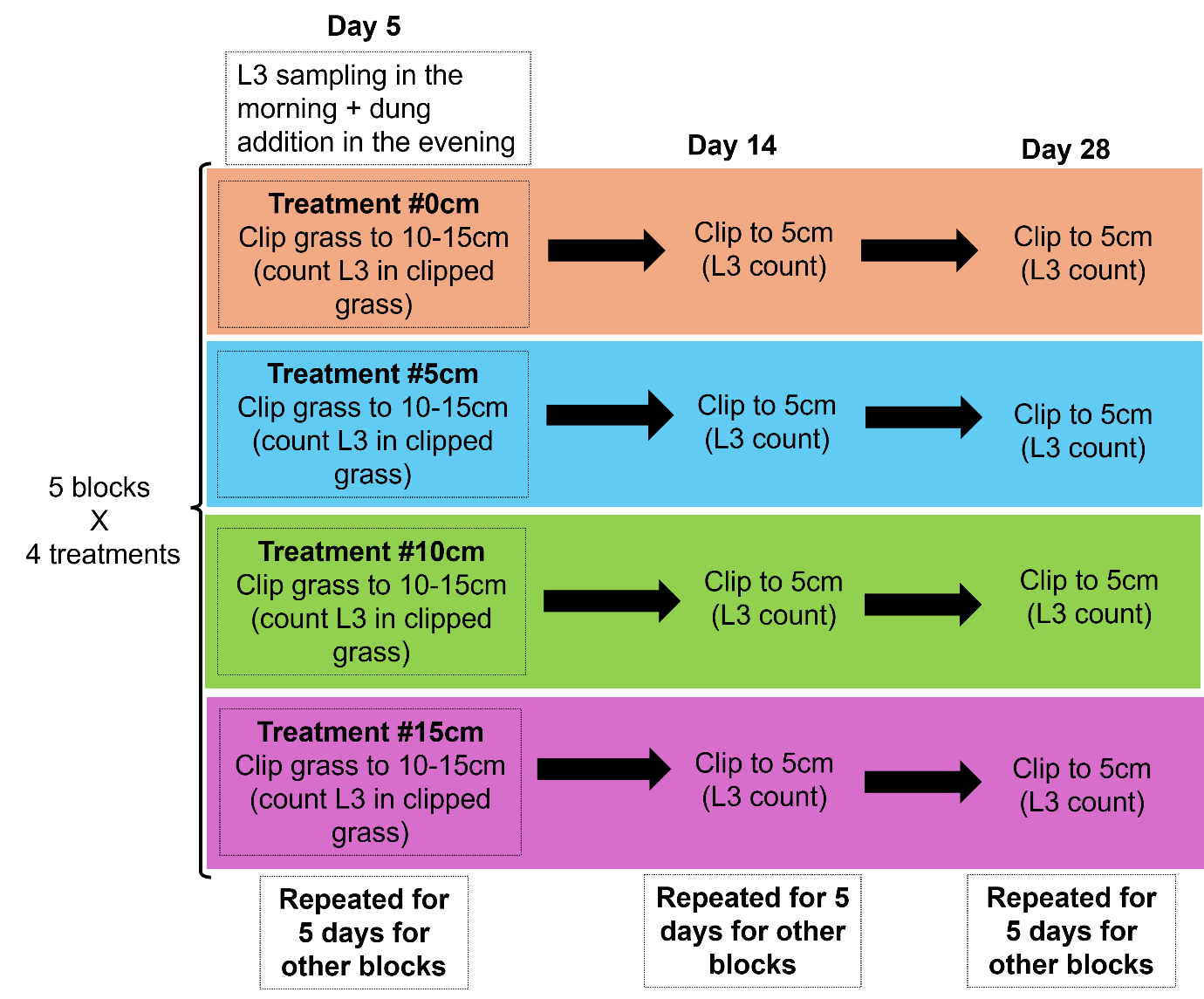
**

**Table S2:** Summary of burial and surface treatments applied in the second experiment

**
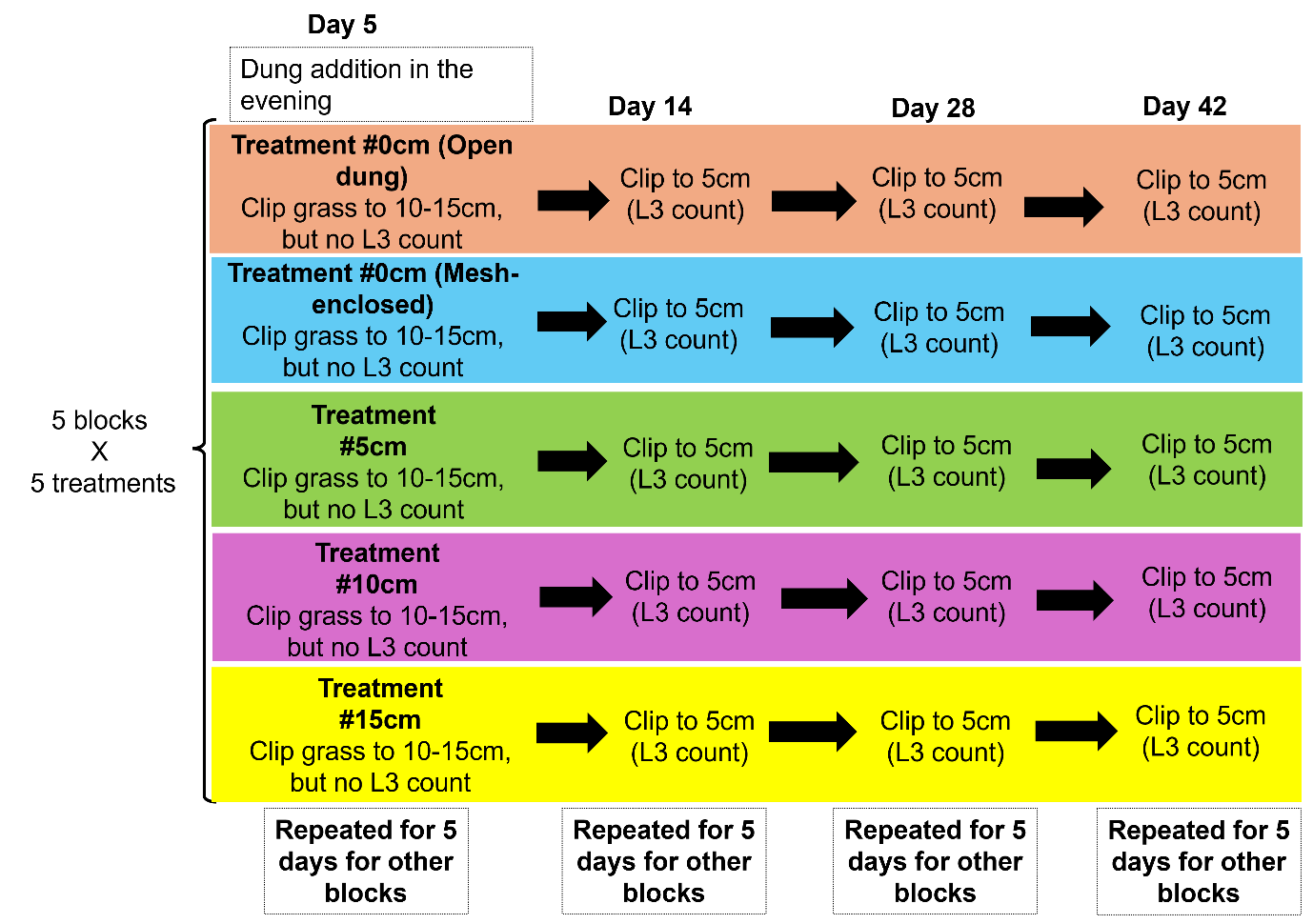
**

## **Table S3:** Number of ruminant larvae (L3) recorded for each burial treatment (0–15 cm and control [C], representing mesh-enclosed dung) across sampling days (0, 14, 28, and 42) in Serengeti National Park during January–February 2024 for Experiment 1 (EXP1_1) and November–December 2024 for Experiment 2 (EXP_2), including dry grass weight (DGW) and mean temperature.

| **CAGE ID** | **Experiment_Block** | **Treatment/cm** | **Day** | **L3 Count** | **DGW** | **Mean temperature** |
| --- | --- | --- | --- | --- | --- | --- |
| 1 | EXP1_1 | 0 | 5 | 0 | 68.25 | 20.8 |
| 2 | EXP1_1 | 5 | 5 | 0 | 75.72 | 20.8 |
| 3 | EXP1_1 | 10 | 5 | 0 | 77.24 | 20.9 |
| 4 | EXP1_1 | 15 | 5 | 0 | 94.65 | 20.8 |
| 5 | EXP1_2 | 0 | 5 | 0 | 58.26 | 20.6 |
| 6 | EXP1_2 | 5 | 5 | 0 | 47.47 | 20.6 |
| 7 | EXP1_2 | 10 | 5 | 0 | 69.71 | 20.5 |
| 8 | EXP1_2 | 15 | 5 | 0 | 48.64 | 20.6 |
| 9 | EXP1_3 | 0 | 5 | 0 | 51.82 | 19.9 |
| 10 | EXP1_3 | 5 | 5 | 0 | 49.75 | 19.9 |
| 11 | EXP1_3 | 10 | 5 | 0 | 54.74 | 19.8 |
| 12 | EXP1_3 | 15 | 5 | 0 | 93.86 | 19.9 |
| 13 | EXP1_4 | 0 | 5 | 0 | 47.97 | 18.5 |
| 14 | EXP1_4 | 5 | 5 | 0 | 82.7 | 18.5 |
| 15 | EXP1_4 | 10 | 5 | 0 | 56.94 | 18.7 |
| 16 | EXP1_4 | 15 | 5 | 0 | 63.87 | 18.5 |
| 17 | EXP1_5 | 0 | 5 | 1 | 117.04 | 20.3 |
| 18 | EXP1_5 | 5 | 5 | 0 | 106.75 | 20.4 |
| 19 | EXP1_5 | 10 | 5 | 0 | 100.31 | 20.2 |
| 20 | EXP1_5 | 15 | 5 | 0 | 103.36 | 20.3 |
| 21 | EXP1_1 | 0 | 14 | 3 | 49.22 | 19.9 |
| 22 | EXP1_1 | 5 | 14 | 1 | 49.27 | 20.1 |
| 23 | EXP1_1 | 10 | 14 | 3 | 37.52 | 20 |
| 24 | EXP1_1 | 15 | 14 | 0 | 50.02 | 20 |
| 25 | EXP1_2 | 0 | 14 | 3 | 38.17 | 21.1 |
| 26 | EXP1_2 | 5 | 14 | 0 | 51.91 | 21.1 |
| 27 | EXP1_2 | 10 | 14 | 10 | 44.5 | 21.2 |
| 28 | EXP1_2 | 15 | 14 | 2 | 38.85 | 21.1 |
| 29 | EXP1_3 | 0 | 14 | 1 | 40.32 | 21.2 |
| 30 | EXP1_3 | 5 | 14 | 5 | 46.25 | 21.2 |
| 31 | EXP1_3 | 10 | 14 | 5 | 35.18 | 21.3 |
| 32 | EXP1_3 | 15 | 14 | 2 | 57.8 | 21.2 |
| 33 | EXP1_4 | 0 | 14 | 4 | 35.55 | 21.1 |
| 34 | EXP1_4 | 5 | 14 | 6 | 45.07 | 21.1 |
| 35 | EXP1_4 | 10 | 14 | 7 | 35.63 | 21.1 |
| 36 | EXP1_4 | 15 | 14 | 5 | 35.09 | 21.2 |
| 37 | EXP1_5 | 0 | 14 | 4 | 52.54 | 21.2 |
| 38 | EXP1_5 | 5 | 14 | 7 | 55.69 | 21.1 |
| 39 | EXP1_5 | 10 | 14 | 6 | 57.54 | 21 |
| 40 | EXP1_5 | 15 | 14 | 2 | 62.41 | 21.1 |
| 41 | EXP1_1 | 0 | 28 | 0 | 35.27 | 21.4 |
| 42 | EXP1_1 | 5 | 28 | 3 | 51.17 | 21.5 |
| 43 | EXP1_1 | 10 | 28 | 2 | 35.31 | 21.4 |
| 44 | EXP1_1 | 15 | 28 | 3 | 47.46 | 21.5 |
| 45 | EXP1_2 | 0 | 28 | 8 | 52.47 | 21.4 |
| 46 | EXP1_2 | 5 | 28 | 13 | 65.86 | 21.4 |
| 47 | EXP1_2 | 10 | 28 | 4 | 54.81 | 21.4 |
| 48 | EXP1_2 | 15 | 28 | 3 | 53.97 | 21.4 |
| 49 | EXP1_3 | 0 | 28 | 4 | 51 | 22.3 |
| 50 | EXP1_3 | 5 | 28 | 9 | 76.19 | 22.2 |
| 51 | EXP1_3 | 10 | 28 | 7 | 60.34 | 22.3 |
| 52 | EXP1_3 | 15 | 28 | 7 | 68.02 | 22.5 |
| 53 | EXP1_4 | 0 | 28 | 2 | 47.09 | 20.7 |
| 54 | EXP1_4 | 5 | 28 | 3 | 48.41 | 20.7 |
| 55 | EXP1_4 | 10 | 28 | 4 | 38.71 | 20.7 |
| 56 | EXP1_4 | 15 | 28 | 3 | 62.3 | 20.8 |
| 57 | EXP1_5 | 0 | 28 | 2 | 67.21 | 20.4 |
| 58 | EXP1_5 | 5 | 28 | 1 | 57.43 | 20.4 |
| 59 | EXP1_5 | 10 | 28 | 2 | 70.31 | 20.5 |
| 60 | EXP1_5 | 15 | 28 | 2 | 69.26 | 20.4 |
| 1 | EXP2_1 | 0 | 14 | 6 | 141.52 | 23.1 |
| 2 | EXP2_1 | 5 | 14 | 75 | 118.53 | 23 |
| 3 | EXP2_1 | 10 | 14 | 59 | 117.55 | 22.4 |
| 4 | EXP2_1 | 15 | 14 | 19 | 105.43 | 22.8 |
| 5 | EXP2_1 | C | 14 | 13 | 130.32 | 15.3 |
| 6 | EXP2_2 | 0 | 14 | 37 | 123.52 | 18.8 |
| 7 | EXP2_2 | 5 | 14 | 65 | 137.45 | 21.7 |
| 8 | EXP2_2 | 10 | 14 | 28 | 85.55 | 21.4 |
| 9 | EXP2_2 | 15 | 14 | 18 | 86.99 | 21.5 |
| 10 | EXP2_2 | C | 14 | 8 | 131.02 | 15 |
| 11 | EXP2_3 | 0 | 14 | 5 | 92.67 | 27.5 |
| 12 | EXP2_3 | 5 | 14 | 25 | 96.58 | 22.1 |
| 13 | EXP2_3 | 10 | 14 | 34 | 94.76 | 22.8 |
| 14 | EXP2_3 | 15 | 14 | 45 | 86.06 | 18.3 |
| 15 | EXP2_3 | C | 14 | 9 | 96.6 | 18.3 |
| 16 | EXP2_4 | 0 | 14 | 19 | 78.92 | 23.9 |
| 17 | EXP2_4 | 5 | 14 | 20 | 113.87 | 23.2 |
| 18 | EXP2_4 | 10 | 14 | 27 | 115.4 | 23.4 |
| 19 | EXP2_4 | 15 | 14 | 3 | 99.91 | 22.8 |
| 20 | EXP2_4 | C | 14 | 6 | 74.66 | 22.8 |
| 21 | EXP2_5 | 0 | 14 | 39 | 107.93 | 22.8 |
| 22 | EXP2_5 | 5 | 14 | 13 | 146.14 | 23.9 |
| 23 | EXP2_5 | 10 | 14 | 30 | 100.96 | 23.9 |
| 24 | EXP2_5 | 15 | 14 | 26 | 110.08 | 22.6 |
| 25 | EXP2_5 | C | 14 | 25 | 123.27 | 22.6 |
| 26 | EXP2_1 | 0 | 28 | 2 | 54.62 | 23.1 |
| 27 | EXP2_1 | 5 | 28 | 6 | 54.7 | 22.6 |
| 28 | EXP2_1 | 10 | 28 | 3 | 40.81 | 23.1 |
| 29 | EXP2_1 | 15 | 28 | 0 | 44.91 | 22.7 |
| 30 | EXP2_1 | C | 28 | 2 | 39.95 | 21.6 |
| 31 | EXP2_2 | 0 | 28 | 0 | 44.27 | 21.6 |
| 32 | EXP2_2 | 5 | 28 | 8 | 53.41 | 21.1 |
| 33 | EXP2_2 | 10 | 28 | 8 | 60.04 | 21.5 |
| 34 | EXP2_2 | 15 | 28 | 6 | 50.44 | 21.8 |
| 35 | EXP2_2 | C | 28 | 7 | 31.66 | 19.5 |
| 36 | EXP2_3 | 0 | 28 | 10 | 81.8 | 19.5 |
| 37 | EXP2_3 | 5 | 28 | 21 | 64.62 | 21.4 |
| 38 | EXP2_3 | 10 | 28 | 16 | 70.07 | 21.4 |
| 39 | EXP2_3 | 15 | 28 | 8 | 69.31 | 16.8 |
| 40 | EXP2_3 | C | 28 | 10 | 65.37 | 16.8 |
| 41 | EXP2_4 | 0 | 28 | 12 | 60.54 | 20.4 |
| 42 | EXP2_4 | 5 | 28 | 20 | 79.25 | 20.4 |
| 43 | EXP2_4 | 10 | 28 | 5 | 80.28 | 20.2 |
| 44 | EXP2_4 | 15 | 28 | 9 | 65.34 | 20.1 |
| 45 | EXP2_4 | C | 28 | 18 | 62.61 | 20.1 |
| 46 | EXP2_5 | 0 | 28 | 29 | 78.58 | 22.3 |
| 47 | EXP2_5 | 5 | 28 | 10 | 66.38 | 21.8 |
| 48 | EXP2_5 | 10 | 28 | 10 | 68.22 | 21.9 |
| 49 | EXP2_5 | 15 | 28 | 26 | 63.73 | 21.9 |
| 50 | EXP2_5 | C | 28 | 15 | 84.98 | 21.9 |
| 51 | EXP2_1 | 0 | 42 | 1 | 91.53 | 23.7 |
| 52 | EXP2_1 | 5 | 42 | 5 | 72.88 | 23 |
| 53 | EXP2_1 | 10 | 42 | 2 | 65.5 | 22.9 |
| 54 | EXP2_1 | 15 | 42 | 6 | 78.6 | 23.3 |
| 55 | EXP2_1 | C | 42 | 7 | 91.48 | 23.3 |
| 56 | EXP2_2 | 0 | 42 | 0 | 90.2 | 23.9 |
| 57 | EXP2_2 | 5 | 42 | 1 | 78.5 | 23.2 |
| 58 | EXP2_2 | 10 | 42 | 1 | 83.87 | 23.2 |
| 59 | EXP2_2 | 15 | 42 | 7 | 92 | 23.3 |
| 60 | EXP2_2 | C | 42 | 7 | 92.76 | 23.3 |
| 61 | EXP2_3 | 0 | 42 | 2 | 67.8 | 23.7 |
| 62 | EXP2_3 | 5 | 42 | 3 | 77.23 | 23.3 |
| 63 | EXP2_3 | 10 | 42 | 5 | 64.85 | 23.1 |
| 64 | EXP2_3 | 15 | 42 | 1 | 85.19 | 18.5 |
| 65 | EXP2_3 | C | 42 | 4 | 66.54 | 18.5 |
| 66 | EXP2_4 | 0 | 42 | 7 | 75.77 | 24.4 |
| 67 | EXP2_4 | 5 | 42 | 3 | 69.69 | 24.4 |
| 68 | EXP2_4 | 10 | 42 | 5 | 67.8 | 23.5 |
| 69 | EXP2_4 | 15 | 42 | 3 | 90.59 | 24.2 |
| 70 | EXP2_4 | C | 42 | 4 | 59.02 | 24.2 |
| 71 | EXP2_5 | 0 | 42 | 10 | 68.16 | 25.5 |
| 72 | EXP2_5 | 5 | 42 | 9 | 84 | 22.7 |
| 73 | EXP2_5 | 10 | 42 | 11 | 66.83 | 23.5 |
| 74 | EXP2_5 | 15 | 42 | 6 | 73.23 | 23.8 |
| 75 | EXP2_5 | C | 42 | 2 | 72.82 | 23.8 |

**Table S4:** Dung removal rate experiment comparing dung left on the surface under open (O) and mesh-enclosed control (C) treatments in Experiment 2.

| **Block** | **Cage id** | **Dung ID** | **Initial weight (g)** | **Remaining weight (g)** | **Removed weight (g)** | **Percentage removed (%)** | **Treatment (open dung (O), mesh-enclosed (C))** | **Hole number (evidence of dung beetle activity)** |
| --- | --- | --- | --- | --- | --- | --- | --- | --- |
| 5 | D17 | 1 | 150 | 92.5 | 57.5 | 38.33 | O | 4 |
| 5 | D17 | 2 | 150 | 84.5 | 65.5 | 43.67 | O | 0 |
| 5 | D17 | 3 | 150 | 94.2 | 55.8 | 37.2 | O | 2 |
| 5 | D17 | 4 | 150 | 90.8 | 59.2 | 39.47 | O | 1 |
| 5 | D17 | 5 | 150 | 90.5 | 59.5 | 39.67 | O | 4 |
| 5 | D17 | 6 | 150 | 85.7 | 64.3 | 42.87 | O | 0 |
| 5 | D17 | 7 | 150 | 80 | 70 | 46.67 | O | 1 |
| 5 | D17 | 8 | 150 | 90.8 | 59.2 | 39.47 | O | 1 |
| 5 | D17 | 9 | 150 | 70.8 | 79.2 | 52.8 | O | 0 |
| 5 | D17 | 10 | 150 | 82.9 | 67.1 | 44.73 | O | 0 |
| 5 | D17 | 11 | 150 | 91.4 | 58.6 | 39.07 | O | 5 |
| 5 | D17 | 12 | 150 | 83 | 67 | 44.67 | O | 3 |
| 5 | D17 | 13 | 150 | 90.5 | 59.5 | 39.67 | O | 2 |
| 5 | D17 | 14 | 150 | 103.1 | 46.9 | 31.27 | O | 1 |
| 5 | D17 | 15 | 150 | 94.6 | 55.4 | 36.93 | O | 1 |
| 5 | X1 | 1 | 150 | 96.8 | 53.2 | 35.47 | C | 0 |
| 5 | X1 | 2 | 150 | 106.6 | 43.4 | 28.93 | C | 0 |
| 5 | X1 | 3 | 150 | 97.6 | 52.4 | 34.93 | C | 0 |
| 5 | X1 | 4 | 150 | 106.5 | 43.5 | 29 | C | 0 |
| 5 | X1 | 5 | 150 | 109.8 | 40.2 | 26.8 | C | 0 |
| 5 | X1 | 6 | 150 | 113.3 | 36.7 | 24.47 | C | 0 |
| 5 | X1 | 7 | 150 | 103.7 | 46.3 | 30.87 | C | 0 |
| 5 | X1 | 8 | 150 | 109.2 | 40.8 | 27.2 | C | 0 |
| 5 | X1 | 9 | 150 | 111.4 | 38.6 | 25.73 | C | 0 |
| 5 | X1 | 10 | 150 | 104.3 | 45.7 | 30.47 | C | 0 |
| 5 | X1 | 11 | 150 | 105.4 | 44.6 | 29.73 | C | 0 |
| 5 | X1 | 12 | 150 | 100.1 | 49.9 | 33.27 | C | 0 |
| 5 | D13 | 13 | 150 | 111.9 | 38.1 | 25.4 | C | 0 |
| 5 | D13 | 14 | 150 | 116.9 | 33.1 | 22.07 | C | 0 |
| 5 | D13 | 15 | 150 | 108.2 | 41.8 | 27.87 | C | 0 |
| 4 | D13 | 1 | 150 | 83.6 | 66.4 | 44.27 | O | 2 |
| 4 | D13 | 2 | 150 | 91.5 | 58.5 | 39 | O | 3 |
| 4 | D13 | 3 | 150 | 91.7 | 58.3 | 38.87 | O | 1 |
| 4 | D13 | 4 | 150 | 88.4 | 61.6 | 41.07 | O | 1 |
| 4 | D13 | 5 | 150 | 78.8 | 71.2 | 47.47 | O | 0 |
| 4 | D13 | 6 | 150 | 86.1 | 63.9 | 42.6 | O | 2 |
| 4 | D13 | 7 | 150 | 88.1 | 61.9 | 41.27 | O | 4 |
| 4 | D13 | 8 | 150 | 80 | 70 | 46.67 | O | 2 |
| 4 | D13 | 9 | 150 | 82.5 | 67.5 | 45 | O | 4 |
| 4 | D13 | 10 | 150 | 90.1 | 59.9 | 39.93 | O | 1 |
| 4 | D13 | 11 | 150 | 36.5 | 113.5 | 75.67 | O | 8 |
| 4 | D13 | 12 | 150 | 80.8 | 69.2 | 46.13 | O | 1 |
| 4 | D13 | 13 | 150 | 80 | 70 | 46.67 | O | 0 |
| 4 | D13 | 14 | 150 | 82.8 | 67.2 | 44.8 | O | 2 |
| 4 | D13 | 15 | 150 | 86.5 | 63.5 | 42.33 | O | 4 |
| 4 | X2 | 1 | 150 | 103.8 | 46.2 | 30.8 | C | 0 |
| 4 | X2 | 2 | 150 | 90.5 | 59.5 | 39.67 | C | 0 |
| 4 | X2 | 3 | 150 | 101.7 | 48.3 | 32.2 | C | 0 |
| 4 | X2 | 4 | 150 | 100.7 | 49.3 | 32.87 | C | 0 |
| 4 | X2 | 5 | 150 | 105.8 | 44.2 | 29.47 | C | 0 |
| 4 | X2 | 6 | 150 | 95.8 | 54.2 | 36.13 | C | 0 |
| 4 | X2 | 7 | 150 | 93.8 | 56.2 | 37.47 | C | 0 |
| 4 | X2 | 8 | 150 | 93.3 | 56.7 | 37.8 | C | 0 |
| 4 | X2 | 9 | 150 | 93.7 | 56.3 | 37.53 | C | 0 |
| 4 | X2 | 10 | 150 | 98.9 | 51.1 | 34.07 | C | 0 |
| 4 | X2 | 11 | 150 | 95.2 | 54.8 | 36.53 | C | 0 |
| 4 | X2 | 12 | 150 | 101.6 | 48.4 | 32.27 | C | 0 |
| 4 | X2 | 13 | 150 | 98.7 | 51.3 | 34.2 | C | 0 |
| 4 | X2 | 14 | 150 | 101.9 | 48.1 | 32.07 | C | 0 |
| 4 | X2 | 15 | 150 | 93.9 | 56.1 | 37.4 | C | 0 |
| 1 | D01 | 1 | 150 | 53.5 | 96.5 | 64.33 | O | 3 |
| 1 | D01 | 2 | 150 | 56 | 94 | 62.67 | O | 4 |
| 1 | D01 | 3 | 150 | 67.9 | 82.1 | 54.73 | O | 1 |
| 1 | D01 | 4 | 150 | 79.8 | 70.2 | 46.8 | O | 1 |
| 1 | D01 | 5 | 150 | 55.9 | 94.1 | 62.73 | O | 3 |
| 1 | D01 | 6 | 150 | 38.6 | 111.4 | 74.27 | O | 3 |
| 1 | D01 | 7 | 150 | 82.3 | 67.7 | 45.13 | O | 0 |
| 1 | D01 | 8 | 150 | 72.6 | 77.4 | 51.6 | O | 3 |
| 1 | D01 | 9 | 150 | 52.8 | 97.2 | 64.8 | O | 5 |
| 1 | D01 | 10 | 150 | 50.2 | 99.8 | 66.53 | O | 1 |
| 1 | D01 | 11 | 150 | 31.7 | 118.3 | 78.87 | O | 2 |
| 1 | D01 | 12 | 150 | 32.6 | 117.4 | 78.27 | O | 0 |
| 1 | D01 | 13 | 150 | 62.8 | 87.2 | 58.13 | O | 2 |
| 1 | D01 | 14 | 150 | 48.6 | 101.4 | 67.6 | O | 1 |
| 1 | D01 | 15 | 150 | 52.6 | 97.4 | 64.93 | O | 3 |
| 1 | X3 | 1 | 150 | 85.1 | 64.9 | 43.27 | C | 0 |
| 1 | X3 | 2 | 150 | 91.4 | 58.6 | 39.07 | C | 0 |
| 1 | X3 | 3 | 150 | 94.6 | 55.4 | 36.93 | C | 0 |
| 1 | X3 | 4 | 150 | 94.4 | 55.6 | 37.07 | C | 0 |
| 1 | X3 | 5 | 150 | 93.1 | 56.9 | 37.93 | C | 0 |
| 1 | X3 | 6 | 150 | 91.9 | 58.1 | 38.73 | C | 0 |
| 1 | X3 | 7 | 150 | 86.5 | 63.5 | 42.33 | C | 0 |
| 1 | X3 | 8 | 150 | 96.7 | 53.3 | 35.53 | C | 0 |
| 1 | X3 | 9 | 150 | 90 | 60 | 40 | C | 0 |
| 1 | X3 | 10 | 150 | 94.9 | 55.1 | 36.73 | C | 0 |
| 1 | X3 | 11 | 150 | 92.5 | 57.5 | 38.33 | C | 0 |
| 1 | X3 | 12 | 150 | 79.6 | 70.4 | 46.93 | C | 0 |
| 1 | X3 | 13 | 150 | 95.2 | 54.8 | 36.53 | C | 0 |
| 1 | X3 | 14 | 150 | 93.7 | 56.3 | 37.53 | C | 0 |
| 1 | X3 | 15 | 150 | 91.2 | 58.8 | 39.2 | C | 0 |
| 2 | D05 | 1 | 150 | 61.7 | 88.3 | 58.87 | O | 1 |
| 2 | D05 | 2 | 150 | 79.7 | 70.3 | 46.87 | O | 0 |
| 2 | D05 | 3 | 150 | 72.2 | 77.8 | 51.87 | O | 1 |
| 2 | D05 | 4 | 150 | 40.3 | 109.7 | 73.13 | O | 3 |
| 2 | D05 | 5 | 150 | 84.3 | 65.7 | 43.8 | O | 1 |
| 2 | D05 | 6 | 150 | 76.4 | 73.6 | 49.07 | O | 2 |
| 2 | D05 | 7 | 150 | 69.3 | 80.7 | 53.8 | O | 4 |
| 2 | D05 | 8 | 150 | 82.6 | 67.4 | 44.93 | O | 2 |
| 2 | D05 | 9 | 150 | 80.8 | 69.2 | 46.13 | O | 2 |
| 2 | D05 | 10 | 150 | 85.8 | 64.2 | 42.8 | O | 1 |
| 2 | D05 | 11 | 150 | 70 | 80 | 53.33 | O | 2 |
| 2 | D05 | 12 | 150 | 77.5 | 72.5 | 48.33 | O | 1 |
| 2 | D05 | 13 | 150 | 100.1 | 49.9 | 33.27 | O | 0 |
| 2 | D05 | 14 | 150 | 61 | 89 | 59.33 | O | 3 |
| 2 | D05 | 15 | 150 | 48.5 | 101.5 | 67.67 | O | 1 |
| 2 | X4 | 1 | 150 | 99.7 | 50.3 | 33.53 | C | 0 |
| 2 | X4 | 2 | 150 | 97 | 53 | 35.33 | C | 0 |
| 2 | X4 | 3 | 150 | 95.1 | 54.9 | 36.6 | C | 0 |
| 2 | X4 | 4 | 150 | 99.2 | 50.8 | 33.87 | C | 0 |
| 2 | X4 | 5 | 150 | 96.9 | 53.1 | 35.4 | C | 0 |
| 2 | X4 | 6 | 150 | 101.4 | 48.6 | 32.4 | C | 0 |
| 2 | X4 | 7 | 150 | 97 | 53 | 35.33 | C | 0 |
| 2 | X4 | 8 | 150 | 96.9 | 53.1 | 35.4 | C | 0 |
| 2 | X4 | 9 | 150 | 92.9 | 57.1 | 38.07 | C | 0 |
| 2 | X4 | 10 | 150 | 103.6 | 46.4 | 30.93 | C | 0 |
| 2 | X4 | 11 | 150 | 106.5 | 43.5 | 29 | C | 0 |
| 2 | X4 | 12 | 150 | 98.4 | 51.6 | 34.4 | C | 0 |
| 2 | X4 | 13 | 150 | 98.1 | 51.9 | 34.6 | C | 0 |
| 2 | X4 | 14 | 150 | 113.5 | 36.5 | 24.33 | C | 0 |
| 2 | X4 | 15 | 150 | 92.5 | 57.5 | 38.33 | C | 0 |
| 3 | D09 | 1 | 150 | 95.5 | 54.5 | 36.33 | C | 3 |
| 3 | D09 | 2 | 150 | 80.8 | 69.2 | 46.13 | C | 1 |
| 3 | D09 | 3 | 150 | 50 | 100 | 66.67 | C | 2 |
| 3 | D09 | 4 | 150 | 80.8 | 69.2 | 46.13 | C | 4 |
| 3 | D09 | 5 | 150 | 65 | 85 | 56.67 | C | 2 |
| 3 | D09 | 6 | 150 | 85.5 | 64.5 | 43 | C | 1 |
| 3 | D09 | 7 | 150 | 81.4 | 68.6 | 45.73 | C | 0 |
| 3 | D09 | 8 | 150 | 78.7 | 71.3 | 47.53 | C | 2 |
| 3 | D09 | 9 | 150 | 73.9 | 76.1 | 50.73 | C | 1 |
| 3 | D09 | 10 | 150 | 71.7 | 78.3 | 52.2 | C | 1 |
| 3 | D09 | 11 | 150 | 97.2 | 52.8 | 35.2 | C | 4 |
| 3 | D09 | 12 | 150 | 34 | 116 | 77.33 | C | 2 |
| 3 | D09 | 13 | 150 | 91.8 | 58.2 | 38.8 | C | 3 |
| 3 | D09 | 14 | 150 | 85.7 | 64.3 | 42.87 | C | 1 |
| 3 | D09 | 15 | 150 | 90.4 | 59.6 | 39.73 | C | 2 |
| 3 | X5 | 1 | 150 | 99.2 | 50.8 | 33.87 | C | 0 |
| 3 | X5 | 2 | 150 | 91.7 | 58.3 | 38.87 | C | 0 |
| 3 | X5 | 3 | 150 | 107.1 | 42.9 | 28.6 | C | 0 |
| 3 | X5 | 4 | 150 | 96.7 | 53.3 | 35.53 | C | 0 |
| 3 | X5 | 5 | 150 | 108.4 | 41.6 | 27.73 | C | 0 |
| 3 | X5 | 6 | 150 | 103.5 | 46.5 | 31 | C | 0 |
| 3 | X5 | 7 | 150 | 110.1 | 39.9 | 26.6 | C | 0 |
| 3 | X5 | 8 | 150 | 105.8 | 44.2 | 29.47 | C | 0 |
| 3 | X5 | 9 | 150 | 101.9 | 48.1 | 32.07 | C | 0 |
| 3 | X5 | 10 | 150 | 110.7 | 39.3 | 26.2 | C | 0 |
| 3 | X5 | 11 | 150 | 108.6 | 41.4 | 27.6 | C | 0 |
| 3 | X5 | 12 | 150 | 102 | 48 | 32 | C | 0 |
| 3 | X5 | 13 | 150 | 105.3 | 44.7 | 29.8 | C | 0 |
| 3 | X5 | 14 | 150 | 110.5 | 39.5 | 26.33 | C | 0 |
| 3 | X5 | 15 | 150 | 101.9 | 48.1 | 32.07 | C | 0 |

**Table S5:** Global model summary output of larval density (a) with interactions, (b) main effects only, and (c) a simplified model. Std = standard

| **Model** | **Term** | **Estimate** | **SE** | **z value** | **P value** |
| --- | --- | --- | --- | --- | --- |
| **(a) Full model (interaction)** | (Intercept) | -1.632 | 0.224 | -7.295 | <0.001*** |
|  | Burial depth (scaled) | 0.009 | 0.111 | 0.084 | 0.933 |
|  | Burial depth² | -0.237 | 0.090 | -2.652 | 0.008** |
|  | Day 28 | -0.483 | 0.158 | -3.066 | 0.002** |
|  | Day 42 | -1.411 | 0.225 | -6.257 | <0.001*** |
|  | Soil temperature | -0.082 | 0.096 | -0.848 | 0.397 |
|  | Burial depth × Day 28 | -0.035 | 0.159 | -0.219 | 0.827 |
|  | Burial depth × Day 42 | 0.036 | 0.201 | 0.181 | 0.857 |
| **(b) Additive model (with temperature)** | (Intercept) | -1.632 | 0.224 | -7.281 | <0.001*** |
|  | Burial depth (scaled) | 0.001 | 0.073 | 0.021 | 0.984 |
|  | Burial depth² | -0.238 | 0.090 | -2.663 | 0.008** |
|  | Day 28 | -0.483 | 0.158 | -3.067 | 0.002** |
|  | Day 42 | -1.405 | 0.225 | -6.249 | <0.001*** |
|  | Soil temperature | -0.086 | 0.096 | -0.901 | 0.368 |
| **(c) Final model (selected)** | (Intercept) | -1.630 | 0.219 | -7.450 | <0.001*** |
|  | Burial depth (scaled) | 0.014 | 0.072 | 0.189 | 0.850 |
|  | Burial depth² | -0.225 | 0.089 | -2.512 | 0.012* |
|  | Day 28 | -0.460 | 0.157 | -2.923 | 0.003** |
|  | Day 42 | -1.482 | 0.210 | -7.041 | <0.001*** |
